# Drugs and products in beekeeping: Efficacy and perspectives of natural alternatives

**DOI:** 10.17221/88/2025-VETMED

**Published:** 2026-05-26

**Authors:** Imrich Szabo, Lucia Sabova, Rastislav Sabo, Monika Sucik

**Affiliations:** ^1^Department of Biology and Physiology, University of Veterinary Medicine and Pharmacy in Kosice, Kosice, Slovak Republic; ^2^Department of Pharmacology and Toxicology, University of Veterinary Medicine and Pharmacy in Kosice, Kosice, Slovak Republic

**Keywords:** integrated bee protection, *Nosema ceranae*, organic acids, plant extracts, veterinary preparations, *Varroa destructor*

## Abstract

Honeybee health is a key factor in sustainable apiculture, with the most significant colony losses attributed to the mite *Varroa destructor* and microsporidia of the genus *Nosema* spp. The aim of this review article is to summarise current knowledge on the use of pharmaceuticals and preparations in the prevention and treatment of these diseases and to highlight promising alternatives to synthetic acaricides. Commonly used active substances such as amitraz and pyrethroids show decreased efficacy in many countries due to the development of resistance. Therefore, organic acids (formic, oxalic, and lactic), essential oils, and plant extracts are increasingly used as environmentally friendly, residue-free alternatives. Some plant preparations and extracts have been shown to be effective against *Nosema ceranae*. In addition to therapeutic interventions, the importance of preventive measures and immune support through supplements containing vitamins, amino acids, and probiotics is emphasised. The article highlights the need for integrated bee health management combining pharmacological, natural, and biotechnological approaches to limit resistance development, minimise residues in bee products, and promote overall colony vitality.

## INTRODUCTION

*Varroa destructor* and *Nosema* spp. are the main biotic factors posing a serious threat to honeybees (Wilfert et al. 2016; Schuler et al. 2023). *Varroa destructor* is a parasitic mite that has spread from the original eastern honeybee (*Apis cerana)* in East Asia to honeybees (De Jong et al. 1982). This mite has become a major problem for beekeepers not only in Slovakia but also worldwide and is considered the most common cause of colony collapse disorder (Rosenkranz et al. 2010; Traynor et al. 2020; Jack and Ellis 2021; De Jong and Lester 2023). Today, beekeepers have a wide range of options available to combat *Varroa* mites, from non-pharmacological biotechnological methods to pharmacotherapy, which involves the use of various drugs, including organic acids (Haber et al. 2019; Jack and Ellis 2021; Szabo 2022; Brodschneider et al. 2023). Approved drugs include the synthetic acaricides amitraz, coumaphos, flumethrin, and tau-fluvalinate. As mites are becoming resistant to the synthetic acaricides, it is necessary to seek new methods of controlling *varroosis* (Thompson et al. 2002; Martin 2004).

In addition to *Varroa* mites, other pathogens have been known to impact bee colonies. On a global scale, microsporidia of the genus *Nosema* spp., are prevalent, with *Nosema apis* (*N.** **apis*) and *Nosema ceranae* (*N.** **ceranae*) being particularly notable species (Fries et al. 1996; Chen et al. 2008; Martin-Hernandez et al. 2012; Emsen et al. 2016; Ansari et al. 2017; Goblirsch 2018; Martin-Hernandez et al. 2018). Currently, fumagillin remains the only registered antibiotic for the treatment of *nosemosis* caused by *N.** **apis* (van den Heever et al. 2014). Several studies have reported its efficacy in the temporary reduction of *N.** **ceranae* spores (Williams et al. 2008; Huang et al. 2013); however, the utilisation is associated with a risk of residue accumulation in bee products and negative impacts on bees (Nozal et al. 2008; Kanda et al. 2011; Huang et al. 2013; van den Heever et al. 2015). Nonetheless, fumagillin is currently restricted in numerous countries (Chaimanee et al. 2021), indicating a pressing need for novel and effective treatments for *nosemosis*. The administration of plant extracts and natural supplements has been studied as a potential treatment for *nosemosis* (Charistos et al. 2015; Stevanovic et al. 2018; Cilia et al. 2020; Mura et al. 2020; Valizadeh et al. 2020; Chen et al. 2021; Nanetti et al. 2021; Shumkova et al. 2021).

## RESISTANCE

Resistance is defined as the ability of a pathogen to survive doses of drugs that are lethal to sensitive pathogens. Mites have repeatedly demonstrated a high adaptive potential to selective pressure, leading to resistance to a wide range of commonly used substances. As a result, resistance to frequently applied acaricides in *Varroa* mites has gradually emerged, including pyrethroids (tau-fluvalinate and flumethrin) and amitraz (Croft and Van De Baan 1988; Lodesani et al. 1995; Milani 1995; Thompson et al. 2002; Martin 2004; Mitton et al. 2022). The extensive use of miticides over the years has exerted strong selective pressure on mite populations, leading to the emergence of resistant populations worldwide (Milani 1995). These resistant populations have demonstrated the capacity to survive doses or concentrations of drugs that formerly induced high rates of mortality (Rinkevich 2020).

Repeated, long-term, and often exclusive use of synthetic miticides has significantly contributed to the development of pharmacoresistance, especially when not used in accordance with label instructions, with insufficient rotation of active ingredients, or with the use of unauthorised domestic preparations (Rinkevich 2024). Resistance thus creates a vicious circle in which reduced sensitivity to miticides leads to their repeated and excessive application of mentioned compounds. Consequently, sensitive mites are eliminated, while the resistant part of the population continues to reproduce and gradually becomes dominant (Maggi et al. 2008; Le Conte et al. 2010; Maggi et al. 2010; Maggi et al. 2011; Gonzalez-Cabrera et al. 2013; Gonzalez-Cabrera et al. 2016).

The gradual development of resistance in *Varroa destructor* has therefore become a multifactorial process, driven not only by selective pressure from long-term miticide use but also by the physiological and molecular mechanisms available to the mite.

Recent genomic analyses have identified specific mutations associated with pyrethroid resistance, particularly the L925V/M substitutions in the *VGSC* gene. Additionally, metabolic resistance mechanisms involving cytochrome P450 enzymes and carboxylesterases have been documented in several *Varroa* populations across Europe and North America (Gonzalez-Cabrera et al. 2013; Vlogiannitis et al. 2021).

## ORGANIC ACIDS

For several decades, various organic acids have been used as effective treatments against *Varroa destructor*. The implementation of “soft chemistry” requires a selection of appropriate substances, monitoring of the effects, testing and optimisation. Application of appropriate treatment using organic acids does not leave residues in hives or bee products. Although organic acids are natural components of bee products, they must not be artificially enriched with them. The most commonly used are formic acid, oxalic acid, and lactic acid, in pure form or as registered medicines and preparations (Table 1) (Pohl 2021).

**Table 1:** List of veterinary medicinal products and preparations intended for bees for the treatment of *varroosis* licensed in Slovakia Veterinary medicines and veterinary preparations for honeybees List of registered veterinary drugs

No.	Name of the veterinary medicinal product	Active substance(s)	Indication(s)	Rp.
1.	API-Bioxal 62 mg/ml, solution for use in beehives	*Acidum oxalicum dihydricum*	Treatment of *Varroa destructor* infestation in honeybees (*Apis mellifera*).	without
2.	API-Bioxal 886 mg/g, powder for use in beehives	*Acidum oxalicum dihydricum*	Treatment of *Varroa destructor* infestation in honeybees (*Apis mellifera*).	without
3.	APIGUARD gel (25% thymol) for use in beehives	*Thymolum*	Treatment against *varroosis* caused by the *Varroa destructor* mite.	Rp.
4.	APILIFE Var – strips for beehives for honeybees	*Thymolum* *Eucalyptietheroleum* *Camphora racemica* *Levomentholum*	Treatment of *varroosis* caused by *Varroa destructor*.	without
5.	APIVAR 500 mg Amitraz, strips for beehives for honeybees	*Amitrazum*	Treatment of *varroosis* caused by the *Varroa destructor* mite sensitive to amitraz in honeybees.	Rp.
6.	APIVARTIN fumigation strip for beehives	*Amitrazum*	Treatment of bee colonies infested with the ectoparasitic mite *Varroa destructor*, between October 1^st^ and March 31^st^. Treatment of swarms and artificial swarms (swarm clusters) with a single application after they have settled.	Rp.
7.	AVARTIN B – 90	*Amitrazum*	Diagnosis and treatment of bee mite infestation.	Rp.
8.	BAYVAROL 3.6 mg/strip for bees	*Flumethrinum*	Diagnosis and treatment of *varroosis* (*Varroa destructor*) in honeybees.	Rp.
9.	Dany’s BienenWohl 39.4 mg/ml powder and solution for dispersion in beehives for honeybees	*Acidum oxalicum*	For the treatment of *varroosis* (*Varroa destructor*) in honeybees (*Apis mellifera*) in colonies without brood.	without
10.	FORMICPRO 68.2 g strips for honeybee hives	*Acidum formicum*	Treatment of *varroosis* caused by the mite *Varroa destructor* in honeybees (*Apis mellifera*).	without
11.	FORMIDOL 40 ml strips for beehives	*Acidum formicum* 85%	*Varroosis*, *nosemosis*, and *ascosphaerosis* in bees. Treatment of bee colonies infested or suspected of being infested with the external mite *Varroa destructor* or the fungi *Nosema* spp. and *Ascosphaera apis*.	without
12.	GABON PF 90 mg strips for beehives	*Tau-fluvalinatum*	*Varroosis*. Treatment of bee colonies infested or suspected of being infested with the external mite *Varroa destructor*, even when there is brood in the colony. It is specifically designed to protect the winter generation of bees in late summer and autumn.	Rp.
13.	M-1 AER 240 mg/ml concentrate for preparing a solution for the therapeutic treatment of bees	*Tau-fluvalinatum*	*Varroosis*. Treatment of bee colonies infested or suspected of being infested with the external mite *Varroa destructor* at a time when the colonies are broodless or have only a small area of capped brood (especially in winter and early spring).	Rp.
14.	OXUVAR 5.7%, 41.0 mg/ml concentrate for solution for honeybees	*Acidum oxalicum*	Treatment of *varroosis* in honeybees (*Apis mellifera*) caused by the *Varroa* mite (*Varroa destructor* – bee mite).	without
15.	OXYBEE 3.4 mg/ml powder and solution for dispersion in beehives for honeybees	*Acidum oxalicum*	For the treatment of *varroosis* (*Varroa destructor*) in honeybees (*Apis mellifera*) in colonies without brood.	without
16.	PolyVar Yellow 275 mg strip for a beehive	*Flumethrin*	For the treatment of *varroosis* in honeybees caused by *Varroa destructor* mites sensitive to flumethrin.	Rp.
17.	THYMOVAR, 15 g Thymol in one strip for the beehive	*Thymolum*	Treatment of *varroosis* (mite infestation) in honeybees (*Apis mellifera*) caused by the mite (*Varroa destructor*).	Rp.
18.	VARIDOL 125 mg/ml solution for the therapeutic treatment of bees	*Amitrazum*	*Varroosis*. Treatment of bee colonies infested or suspected of being infested with the ectoparasitic mite *Varroa destructor* at a time when the colonies are broodless or have only a small area of capped brood. Type of treatment: diagnosis and treatment between October 1^st^ and April 15^th^.	Rp.
19.	VarroMed 5 mg/ml + 44 mg/ml dispersion into the hive for honeybees	*Acidum formicum* *Acidum oxalicum*	Treatment of *varroosis* (*Varroa destructor*) in honeybee colonies with and without brood.	without
20.	VarroMed 75 mg + 660 mg dispersion into the hive for honeybees	*Acidum formicum* *Acidum oxalicum*	Treatment of *varroosis* (*Varroa destructor*) in honeybee colonies with and without brood.	without

Source: Institute for State Control of Veterinary Biological and Medicines Nitra 2021 (http://www.uskvbl.sk/; cited July 7, 2025)

Rp. = prescription (required)

In addition to organic acids, essential oils, such as thymol, are also registered for use in bee colonies. Although they can easily penetrate wax, careful use and hygiene of the combs can significantly limit this process (Ritter 2018; Pohl 2021).

## FORMIC ACID

Formic acid is a natural component of many bee products, including honey. This acid, secreted by ants, is used by birds to eliminate mites from their feathers (Ritter 2018). Formic acid was among the first substances tested for the control of *varroosis* and is therefore available in various application forms. The application forms are systems with absorbent carriers that release acid, which evaporates and causes toxic damage to the mite. The primary effect is achieved on females of *Varroa destructor* present on bees, although this acid has also been shown to have a partial effect on mites present in/on capped brood (vanEngelsdorp et al. 2008). It causes damage to *Varroa destructor* females, which are unable to mate, or damage to mite development in the brood cell. Formic acid also significantly suppresses *nosemosis*. In 1992, experiments conducted at the Výzkumný ústav včelařský (VÚVč) in Dol demonstrated up to 95% effectiveness against *Nosema* spp. spores in the hive environment when exposed to an evaporation plate with 85% formic acid for 4** **days (Titera 2017).

There are two main approaches to utilisation:

- short-term evaporation systems;
- long-term evaporation systems.

When applying the product, the stages of brood development must be considered to ensure an effective evaporation effect. These conditions are met by long-term evaporators; for short-term evaporators, the application must be repeated (PSNV CZ 2016). To date, experience with formic acid indicates a better treatment effect in heavily concentrated bee colonies. Thus, the most suitable time for treatment is after the honeycomb has been removed and preparations for winter have been made (Ritter 2018).

### Short-term evaporation systems

The effect of short-term evaporators lasts only 2–4** **days. If a capped brood is present in the colony, the application must be repeated. Both 85% and 60% formic acid evaporators are used. Their advantages often include low treatment costs and, in some cases, dose variability across individual colonies. The disadvantage may be greater labour intensity (PSNV CZ 2016).

### Long-term evaporation systems

The aim of using long-term evaporation systems is to kill as many females of *Varroa destructor* in the colony as possible in the presence of capped brood with a single treatment. Therefore, long-term evaporators are designed for effective evaporation lasting at least 10** **days. There are also evaporators that allow the active substance to evaporate for 2–3** **weeks. In the Middle Europe region, the most commonly used evaporators are the Nassenheider, Yannick, Mitegone, and Liebig evaporators, available in various modifications (PSNV CZ 2016; Pohl 2021).

The application of each treatment must be considerate, as it can impose a significant burden on the bee organism. Damage to the brood can occur mainly in the vicinity of a saturated sponge carrier or evaporator** **– bees remove damaged/dead brood. Newly hatched bees are also sensitive to acids. During the treatment process, 50 to 100** **young bees may die, which is considered acceptable, as the colony can quickly replace this loss. Losses of the queen are rare but possible (Pohl 2021).

The available literature has not yet demonstrated the emergence of resistance to formic acid in* Varroa destructor*, despite its use for approximately four decades. Most studies report an efficacy rate of over 70%, with lower values typically attributable to disparities in methodology and experimental conditions. The key factor for success is the actual rate of acid evaporation, which fundamentally affects the outcome of the treatment. Therefore, the need for accurate monitoring of evaporation and, ideally, the concentration of acid in the hive air during application is emphasised. However, the overall variability of the data necessitates reporting median efficacy, while the available results do not indicate the presence of resistance (Kosch et al. 2025).

## OXALIC ACID

Oxalic acid is a widely distributed compound in nature. Its presence has also been identified in plants such as sorrel, rhubarb, and spinach. Oxalic acid is harmful to the body, especially in the form of crystals. Spraying and evaporation as methods of application are not permitted in some countries, or are permitted only with the use of safety pads. Globally approved methods of application include dripping and spraying with a 3.5% oxalic acid dihydrate solution, as well as sublimation. The most commonly used treatments involving organic acids are sublimation and dripping a sugar solution of oxalic acid onto bees (Ritter 2018; Pohl 2021).

The sublimation method requires technical equipment, i.e., a device that heats crystalline acid, causing it to sublimate. Protective equipment, especially a respirator, must be used when treating with the sublimation method. Due to its ability to bind calcium in the body and to form insoluble oxalates, it is recommended to treat the bees with oxalic acid only once. If the treatment is carried out on the summer bee colony, the winter treatment can also be performed, provided that a different generation of bees is present in the colony (PSNV CZ 2016).

The application by dripping is also carried out only once, never twice (loss of bees) or three times (death of the colony). The most commonly used concentration of oxalic acid solution is 3.5% to 4.5% applied in sugar syrup by dripping directly onto the bees present between the frames (PSNV CZ 2016; Pohl 2021). Oxalic acid can also be applied to treat swarms, clusters, or young colonies without capped brood (PSNV CZ 2016).

Although oxalic acid is generally regarded as one of the most reliable organic treatments, its biological effects on bees can vary depending on the method of application.

The study by Pindakova et al. (2025) provides important insights into how different acaricidal treatments affect honeybee physiology and immunity. Treatment with oxalic acid – glycerine strips (OA-G) resulted in a significant increase in antimicrobial activity in the haemolymph and stimulated the production of several antimicrobial peptides, indicating a strong immune response in the bees. Such activation may be beneficial in the defence against pathogens; however, long-term stimulation of immunity may also be detrimental and lead to energetic costs or tissue damage. Because the experiment was conducted over a short period, it was not possible to assess the long-term biological impacts of these treatments. Compared to oxalic acid – trickling (OA-T) treatment, OA-G has a smaller effect on vitellogenin levels and overall physiology of the bees. Although OA-T induced a short-term increase in vitellogenin levels, further exploration is still required.

## LACTIC ACID

Lactic acid, like oxalic acid and formic acid, is found in many foods. In honey, it is mainly present in the L(+) form, which is particularly valuable to humans. A mixture of its L(+) and D(–) forms, which is commercially available, is used to combat *varroosis* (Ritter 2018). Treatment with lactic acid is one alternative to synthetic acaricides for the treatment of bee colonies with *varroosis*. It is an effective method of reducing the number of mites in a bee colony. The most commonly used method is to apply a 15% lactic acid solution directly to adult bees as an aerosol via a sprayer. The disadvantage is the need to dismantle the entire colony and treat the bees on the comb frame by frame. This obviously requires more time to treat a single colony and is very labour-intensive. Due to its labour intensity, this treatment is not suitable for productive colonies but is ideal for treating swarm colonies. The most suitable time to treat swarms is during the phase without a capped brood. If the treatment is carried out without the presence of capped brood, it is highly effective, as all the *Varroa destructor* females are present on the bees and are affected by the acid (PSNV CZ 2016). Outdoor temperatures should not fall below 6** °**C when applying lactic acid (Ritter 2018).

## SYNTHETIC ACARICIDES

Synthetic acaricides, also known as “hard chemicals”, are complex chemical compounds used in beekeeping** **– synthetic active substances that have been used to control *varroosis* since the 1980s (PSNV CZ 2016; Pohl 2021). They have gained popularity among beekeepers worldwide due to their ease of use, but they are detrimental to bees’ health. The first signs of the emerging mite populations resistant to these products have begun to appear relatively early. Resistant populations are a global problem in the control of *varroosis*. In some countries, the level of mite resistance to these products has become so high that their use is ineffective (Bahreini et al. 2025). There is a global trend towards reducing the use of synthetic acaricides, partly due to contamination and the long-term persistence of these drugs in bee products, especially wax. Although this contamination is minimal when utilised correctly, an increasing number of beekeepers are opting to terminate their use. Moreover, cross-resistance has been demonstrated for the pyrethroid group (tau-fluvalinate, flumethrin) and amitraz. This means that if there is resistance to one active substance in this group, other substances are often ineffective due to their similar structure (PSNV CZ 2016).

Current control strategies increasingly rely on Integrated Pest Management (IPM), combining biological, mechanical, and chemical tools. Recommended practice includes monitoring infestation levels, annually rotating active substances, integrating brood interruption, and minimising the use of synthetic acaricides to slow resistance evolution (Jack and Ellis 2021).

One of the major factors causing bee decline is *nosemosis*, which weakens the immune system of bee colonies and contributes to their gradual collapse. Infection by pathogens of the genus Nosema spp. can cause widespread problems in bee colonies, subsequently affecting the overall health and productivity of the colonies.

So far, the only effective treatment against *nosemosis* has been fumagillin, a mycotoxin isolated from *Aspergillus fumigatus* (Williams et al. 2008; Higes et al. 2011). However, treatment with fumagillin can contribute to the development of resistant strains of *N.** **ceranae* and the presence of residues in honey. In addition, it may pose food safety concerns due to its compound toxicity to mammals. Treatment with fumagillin may also affect bee physiology and promote the development of parasites (Huang et al. 2013). Furthermore, this compound is not legally available in all countries. Therefore, finding an alternative to fumagillin is crucial for honeybee health and for preventing environmental problems. The promotion of optimal health in bee colonies enhances resistance to pathogens, thereby positively impacting overwintering success and reducing colony mortality. Therefore, in recent years, increased attention has been paid to the use of natural substances and alternative preparations to control infections caused by *Nosema ceranae*. A study by Chaimanee et al. (2021) evaluated the toxicity and efficacy of 12** **plant extracts, nine of which exhibited high antimicrosporidian activity comparable to that of fumagillin. The most promising extracts (*Annona squamosa*, *Ocimum basilicum*, *Psidium guajava*, and *Syzygium jambos*) significantly reduced spore counts without adverse effects on bee colonies. Similarly, Cilia et al. (2020) demonstrated that treatment with ApiHerb^®^ and Api-Bioxal^®^ reduced the abundance of *N.** **ceranae*, and ApiHerb^®^ also reduced the prevalence of infected bees. An alternative approach was proposed by Arismendi et al. (2018), who showed that methanol extracts from Chilean plants (*Aristotelia chilensis*, *Ugni molinae*, and *Gevuina avellana*) and propolis significantly reduced the burden caused by *N.** **ceranae*, with some of them also promoting the survival of infected bees. Table 2 lists products that promote bee colony vitality.

**Table 2:** List of approved veterinary products for the support of bees in Slovakia List of approved veterinary products

No.	Name of veterinary medicinal product	Active substance(s)	Indication(s)	Rp.
1.	Apibiovit	Vitamin A Vitamin D3 Vitamin E Vitamin B1 Vitamin B2 Vitamin B5 Vitamin B3 Vitamin B6 Vitamin B12 Vitamin H Vitamin K3 Folic acid Choline chloride Methionine Lysine Valine Isoleucine Leucine Serine Glycine Histidine Arginine Threonine Alanine Proline Tyrosine Hydroxylysine Phenylalanine Aspartic acid Glutamic acid Hydroxyproline Tryptophan Inositol	Stimulation of bee colony development in spring and in preparation for wintering. Emergency feeding, especially during periods of nectar shortage and in winter. Stimulation of queen egg laying and brood rearing. The product can be used to feed colonies intended for queen rearing, to accelerate colony development after swarming and merging of bee colonies, and during relocation. The product can be particularly effective before, during, and after the beekeeping season, when pollen and nectar supplies are naturally scarce.	without
2.	Apidez	Thymol Fir essential oil	For the prevention and support of the treatment of *varroosis* in bees.	without
3.	Apilac	*Lactobacillus acidophilus Streptococcus faecium* Vitamin B1 Vitamin B2 Vitamin B3 Vitamin B5 Vitamin B6 Vitamin C	Supplementation of vitamins and natural microflora in bee honey and in the digestive tract of bees. In addition, for feeding bees with sugar syrup, honey cake and royal jelly substitutes and in winter feeding. Increasing immunity against infectious diseases of the brood and adult bees. Restoration of natural microflora after poisoning with chemical sprays that pass through the digestive tract. Colonisation of the environment with lactobacilli after relocation and merging of swarms.	without
4.	Apilekar	Natural honey Powdered sugar Garlic extract Perga extract	Concentrated feed for bees to increase their immunity to adverse conditions and stimulate development and vitality.	without
5.	Bisanar	Thymol Coriander oil Fir essential oil	For the prevention and support of the treatment of *varroosis* in bees.	without
6.	Ekofytol	Needle extract Garlic oil Sea salt	Designed to stimulate the growth and development of bee colonies, increase the resistance of bees to adverse environmental influences, and contribute to increased queen fertilisation and colony vitality.	without
7.	Ekopol	*Artemisia absinthium* essential oil Coriander essential oil Thyme essential oil Menthol essential oil	Acaricidal and repellent element against *Varroa*, *acarapidosis*, and honeycomb weevil.	without
8.	Ekovartin	Coriander essential oil Thyme essential oil Oregano essential oil Peppermint essential oil	Ecological veterinary product for the prevention and support of treatment of *Varroa* and *acarapidosis* in bees.	without
9.	Harmónia prírody	Succinic acid Ascorbic acid Cobalt chloride Garlic Sea salt Glucose	Veterinary preparation for eliminating toxins in bee poisoning of various causes, increasing immunity and stimulating the development of bee colonies.	without
10.	Nozetom	Sea salt Glucose Garlic extract (dry) Ascorbic acid	Veterinary product for bees to increase bees‘ resistance to diseases and adverse environmental influences.	without

Source: Institute for State Control of Veterinary Biologicals and Medicines Nitra 2021 (http://www.uskvbl.sk/; cited July 7, 2025)

Rp. = prescription (required)

## CONCLUSION

This study focused on the use of treatments, especially individual organic acids in commercially available products, and their methods of application. Acknowledgement of the health challenges in modern beekeeping is essential, as mites and associated diseases have been identified as major contributors to bee colony health.

Furthermore, chemical treatments, whether used for prevention or therapy, have been shown to significantly affect the bee organism and the bee colony. Additionally, the presence of residues can impact the quality of bee products.

Considering the escalating resistance exhibited by mites to synthetic substances, it is important to adopt a positive approach to the utilisation of prevention and therapy. The review lists drugs containing organic acids licensed in Slovakia that could be suitable alternatives to synthetic acaricides.

Promising novel approaches include RNA interference targeting essential *Varroa* genes, lithium salts, and microbiome-based interventions. Although none of these methods are ready for broad implementation, they represent important research directions in post-synthetic management of *varroosis.*

Despite the widespread use of fumagillin abroad, pathogens such as *N.** **ceranae* can be effectively controlled using sustainable methods. However, the treatments mentioned in this study were not initially developed for *N.** **ceranae* infection. Further research is needed to expand their application.

Given the increasing incidence of *nosemosis* (*N.** **ceranae*) worldwide, there is an urgent need for safe veterinary treatments to effectively manage this disease. In this context, standardisation of protocols with guidelines for the development of such products is an obvious requirement. Until now, such guidance has only existed for the registration of medicines for the treatment of *Varroa* mite infestation (European Medicines Agency 2011). As honeybee health is becoming an increasingly serious issue in terms of food safety, extending these standards to other honeybee diseases appears to be necessary. Future research focused on the development of integrated treatment approaches will provide us with a comprehensive view of honeybee health.

Moreover, considering that people’s environmental awareness is currently on the rise, this study aims to contribute to the current topic of *Nosema* spp. infection and the *Varroa destructor* population with regard to the alternative approaches and environmental responsibility.

## References

[R1] Ansari MJ, Al-Ghamdi A, Nuru A, Khan KA, Alattal Y. Geographical distribution and molecular detection of Nosema ceranae from indigenous honey bees of Saudi Arabia. Saudi J Biol Sci. 2017 Jul;24(5):983-91. 28663692 10.1016/j.sjbs.2017.01.054 PMC5478367

[R2] Arismendi N, Vargas M, Lopez MD, Barria Y, Zapata N. Promising antimicrobial activity against the honey bee parasite Nosema ceranae by methanolic extracts from Chilean native plants and propolis. J Apic Res. 2018;57(4):522-35.

[R3] Bahreini R, Gonzalez-Cabrera J, Hernandez-Rodriguez CS, Moreno-Marti S, Muirhead S, Labuschagne RB, Rueppell O. Arising amitraz and pyrethroids resistance mutations in the ectoparasitic Varroa destructor mite in Canada. Sci Rep. 2025 Jan 10;15(1):1587. 39794392 10.1038/s41598-025-85279-6 PMC11724071

[R4] Brodschneider R, Schlagbauer J, Arakelyan I, Ballis A, Brus J, Brusbardis V, Cadahia L, Charriere JD, Chlebo R, et al.. Spatial clusters of Varroa destructor control strategies in Europe. J Pest Sci. 2023 Mar;96(2):759-83.

[R5] Chaimanee V, Kasem A, Nuanjohn T, Boonmee T, Siangsuepchart A, Malaithong W, Sinpoo C, Disayathanoowat T, Pettis JS. Natural extracts as potential control agents for Nosema ceranae infection in honeybees, Apis mellifera. J Invertebr Pathol. 2021 Nov;186:107688. 34728218 10.1016/j.jip.2021.107688

[R6] Charistos L, Parashos N, Hatjina F. Long term effects of a food supplement HiveAlive^TM^ on honey bee colony strength and Nosema ceranae spore counts. J Apic Res. 2015;54(5):420-6.

[R7] Chen X, Wang S, Xu Y, Gong H, Wu Y, Chen Y, Hu F, Zheng H. Protective potential of Chinese herbal extracts against microsporidian Nosema ceranae, an emergent pathogen of western honey bees, Apis mellifera L. J Asia Pac Entomol. 2021;24(2):502-12.

[R8] Chen Y, Evans JD, Smith IB, Pettis JS. Nosema ceranae is a long-present and wide-spread microsporidian infection of the European honey bee (Apis mellifera) in the United States. J Invertebr Pathol. 2008 Feb;97(2):186-8. 17880997 10.1016/j.jip.2007.07.010

[R9] Cilia G, Garrido C, Bonetto M, Tesoriero D, Nanetti A. Effect of Api-Bioxal^®^ and ApiHerb^®^ Treatments against Nosema ceranae infection in Apis mellifera investigated by two qPCR methods. Vet Sci. 2020 Sep 4;7(3):125. 32899611 10.3390/vetsci7030125 PMC7558000

[R10] Croft BA, Van De Baan HE. Ecological and genetic factors influencing evolution of pesticide resistance in tetranychid and phytoseiid mites. Exp Appl Acarol. 1988;4(3):277-300.

[R11] De Jong D, Lester PJ. The global challenge of improving bee protection and health. Front Bee Sci. 2023 Feb;1:1118292.

[R12] De Jong D, Morse RA, Eickwort GC. Mite pests of honey bees. Annu Rev Entomol. 1982;27(1):229-52.

[R13] Emsen B, Guzman-Novoa E, Hamiduzzaman MM, Eccles L, Lacey B, Ruiz-Perez RA, Nasr M. Higher prevalence and levels of Nosema ceranae than Nosema apis infections in Canadian honey bee colonies. Parasitol Res. 2016 Jan;115(1):175-81. 26358102 10.1007/s00436-015-4733-3

[R14] European Medicines Agency. Guideline on veterinary medicinal products controlling Varroa destructor parasitosis in bees [Internet]. 2011 [cited 2025 Oct]. Available from: https://www.ema.europa.eu/en/documents/scientific-guideline/guideline-veterinary-medicinal-products-controlling-varroa-destructor-parasitosis-bees_en.pdf.

[R15] Fries I, Feng F, da Silva A, Slemenda SB, Pieniazek NJ. Nosema ceranae n. sp. (Microspora, Nosematidae), morphological and molecular characterization of a microsporidian parasite of the Asian honey bee Apis cerana (Hymenoptera, Apidae). Eur J Protistol. 1996;32(3):356-65.

[R16] Goblirsch M. Nosema ceranae disease of the honey bee (Apis mellifera). Apidologie. 2018 Feb;49(1):131-50.

[R17] Gonzalez-Cabrera J, Davies TG, Field LM, Kennedy PJ, Williamson MS. An amino acid substitution (L925V) associated with resistance to pyrethroids in Varroa destructor. PLoS One. 2013 Dec 18;8(12):e82941. 24367572 10.1371/journal.pone.0082941 PMC3867425

[R18] Gonzalez-Cabrera J, Rodriguez-Vargas S, Davies TG, Field LM, Schmehl D, Ellis JD, Krieger K, Williamson MS. Novel mutations in the voltage-gated sodium channel of pyrethroid-resistant Varroa destructor populations from the Southeastern USA. PLoS One. 2016 May 18;11(5):e0155332. 27191597 10.1371/journal.pone.0155332 PMC4871586

[R19] Haber AI, Steinhauer NA, vanEngelsdorp D. Use of chemical and nonchemical methods for the control of Varroa destructor (Acari: Varroidae) and associated winter colony losses in U.S. beekeeping operations. J Econ Entomol. 2019 Aug 3;112(4):1509-25. 31008501 10.1093/jee/toz088

[R20] Higes M, Nozal MJ, Alvaro A, Barrios L, Meana A, Martin-Hernandez R, Bernal JL, Bernal J. The stability and effectiveness of fumagillin in controlling Nosema ceranae (Microsporidia) infection in honey bees (Apis mellifera) under laboratory and field conditions. Apidologie. 2011;42(4):364-77.

[R21] Huang WF, Solter LF, Yau PM, Imai BS. Nosema ceranae escapes fumagillin control in honey bees. PLoS Pathog. 2013 Mar;9(3):e1003185. 23505365 10.1371/journal.ppat.1003185 PMC3591333

[R22] Institute for State Control of Veterinary Biologicals and Medicines Nitra [Internet]. Nitra: USKVBL; 2021 [cited 2025 Jul]. Available from: http://www.uskvbl.sk/.

[R23] Jack CJ, Ellis JD. Integrated pest management control of Varroa destructor (Acari: Varroidae), the most damaging pest of [Apis mellifera L. (Hymenoptera: Apidae)] Colonies. J Insect Sci. 2021 Sep 1;21(5):6. 10.1093/jisesa/ieab058 PMC8449538 34536080

[R24] Kanda M, Sasamoto T, Takeba K, Hayashi H, Kusano T, Matsushima Y, Nakajima T, Kanai S, Takano I. Rapid determination of fumagillin residues in honey by liquid chromatography-tandem mass spectrometry using the QuEChERS method. J AOAC Int. 2011 May;94(3):878-85. 21797017

[R25] Kosch Y, Mulling C, Emmerich IU. Assessment of resistance of Varroa destructor to formic and lactic acid treatment – A systematic review. Vet Sci. 2025 Feb 8;12(2):144. 40005903 10.3390/vetsci12020144 PMC11860394

[R26] Le Conte Y, Ellis M, Ritter W. Varroa mites and honey bee health: Can Varroa explain part of the colony losses? Apidologie. 2010;41(3):353-63.

[R27] Lodesani M, Colombo M, Spreafico M. Ineffectiveness of Apistan^®^ treatment against the mite Varroa jacobsoni Oud in several districts of Lombardy (Italy). Apidologie. 1995;26(1):67-72.

[R28] Maggi MD, Ruffinengo SR, Gende LB, Eguaras MJ, Sardella NH. LC50 baseline levels of amitraz, coumaphos, fluvalinate and flumethrin in populations of Varroa destructor from Buenos Aires Province, Argentina. J Apic Res. 2008;47(4):292-5.

[R29] Maggi MD, Ruffinengo SR, Negri P, Eguaras MJ. Resistance phenomena to amitraz from populations of the ectoparasitic mite Varroa destructor of Argentina. Parasitol Res. 2010 Oct;107(5):1189-92. 20668878 10.1007/s00436-010-1986-8

[R30] Maggi MD, Ruffinengo SR, Mendoza Y, Ojeda P, Ramallo G, Floris I, Eguaras MJ. Susceptibility of Varroa destructor (Acari: Varroidae) to synthetic acaricides in Uruguay: Varroa mites’ potential to develop acaricide resistance. Parasitol Res. 2011 Apr;108(4):815-21. 20978789 10.1007/s00436-010-2122-5

[R31] Martin SJ. Acaricide (pyrethroid) resistance in Varroa destructor. Bee World. 2004;85(4):67-9.

[R32] Martin-Hernandez R, Bartolome C, Chejanovsky N, Le Conte Y, Dalmon A, Dussaubat C, Garcia-Palencia P, Meana A, Pinto MA, Soroker V, Higes M. Nosema ceranae in Apis mellifera: A 12 years postdetection perspective. Environ Microbiol. 2018 Apr;20(4):1302-29. 29575513 10.1111/1462-2920.14103

[R33] Martin-Hernandez R, Botias C, Bailon EG, Martinez-Salvador A, Prieto L, Meana A, Higes M. Microsporidia infecting Apis mellifera: Coexistence or competition. Is Nosema ceranae replacing Nosema apis? Environ Microbiol. 2012 Aug;14(8):2127-38. 22176602 10.1111/j.1462-2920.2011.02645.x

[R34] Milani N. The resistance of Varroa jacobsoni Oud to pyrethroids: A laboratory assay. Apidologie. 1995;26(5):415-29.

[R35] Mitton GA, Meroi Arcerito F, Cooley H, Fernandez de Landa G, Eguaras MJ, Ruffinengo SR, Maggi MD. More than sixty years living with Varroa destructor: A review of acaricide resistance. Int J Pest Manag. 2022;72(1):1-18.

[R36] Mura A, Pusceddu M, Theodorou P, Angioni A, Floris I, Paxton RJ, Satta A. Propolis consumption reduces Nosema ceranae infection of European honey bees (Apis mellifera). Insects. 2020 Feb 15;11(2):124. 32075232 10.3390/insects11020124 PMC7074184

[R37] Nanetti A, Ugolini L, Cilia G, Pagnotta E, Malaguti L, Cardaio I, Matteo R, Lazzeri L. Seed meals from Brassica nigra and Eruca sativa control artificial Nosema ceranae infections in Apis mellifera. Microorganisms. 2021 Apr 28;9(5):949. 33924845 10.3390/microorganisms9050949 PMC8146933

[R38] Nozal MA, Bernal JL, Martin MA, Bernal J, Alvaro A, Martin R, Higes M. Trace analysis of fumagillin in honey by liquid chromatography-diode array-electrospray ionization mass spectrometry. J Chromatogr A. 2008 May 9;1190(1-2):224-31. 18371976 10.1016/j.chroma.2008.03.019

[R39] Pindakova E, Dostalkova S, Jemelkova J, Furstova J, Hurychova J, Hyrsl P, Titera D, Petrivalsky M, Dobes P, Danihlik J. Enhanced immune response and antimicrobial activity in honey bees (Apis mellifera) following application of oxalic acid-glycerine strips. Pestic Biochem Physiol. 2025 Apr;209:106353. 40082044 10.1016/j.pestbp.2025.106353

[R40] Pohl F. Choroby vciel – Prevencia, diagnostika a liecenie [Bee diseases – Prevention, diagnosis and treatment]. 1^st^ ed. Bratislava: Priroda; 2021. Slovak.

[R41] PSNV CZ. Vcelarstvi [Beekeeping]. 1^st^ ed. Ceske Budejovice: Pracovni spolecnost nastavkovych vcelaru CZ; 2016. Czech.

[R42] Rinkevich FD. Experimental parameters affecting the outcomes of amitraz resistance testing in Varroa destructor. J Apic Res. 2024;63(2):341-9.

[R43] Rinkevich FD. Detection of amitraz resistance and reduced treatment efficacy in the Varroa Mite, Varroa destructor, within commercial beekeeping operations. PLoS One. 2020 Jan 17;15(1):e0227264. 31951619 10.1371/journal.pone.0227264 PMC6968863

[R44] Ritter W. Zdrave vcely – Prevencia, diagnostika a liecba chorob [Healthy bees – Prevention, diagnosis and treatment of diseases]. Bratislava: Citadella; 2018. Slovak.

[R45] Rosenkranz P, Aumeier P, Ziegelmann B. Biology and control of Varroa destructor. J Invertebr Pathol. 2010 Jan;103(Suppl_1):S96-119. 19909970 10.1016/j.jip.2009.07.016

[R46] Schuler V, Liu YC, Gisder S, Horchler L, Groth D, Genersch E. Significant, but not biologically relevant: Nosema ceranae infections and winter losses of honey bee colonies. Commun Biol. 2023 Mar 1;6(1):229. 36859713 10.1038/s42003-023-04587-7 PMC9977864

[R47] Shumkova R, Balkanska R, Hristov P. The herbal supplements NOZEMAT HERB^®^ and NOZEMAT HERB PLUS^®^: An alternative therapy for N. ceranae infection and its effects on honey bee strength and production traits. Pathogens. 2021 Feb 19;10(2):234. 33669663 10.3390/pathogens10020234 PMC7922068

[R48] Stevanovic J, Stanimirovic Z, Simeunovic P, Lakic N, Radovic I, Sokovic M, Griensven LJLDV. The effect of Agaricus brasiliensis extract supplementation on honey bee colonies. An Acad Bras Cienc. 2018 Jan-Mar;90(1):219-29. 29424386 10.1590/0001-3765201820150182

[R49] Szabo I. Ucinok organickych kyselin na populaciu Varroa destructor [Effect of organic acids on the population of Varroa destructor] [diploma thesis]. Kosice: Univerzita veterinarskeho lekarstva a farmacie; 2022. Slovak.

[R50] Thompson HM, Brown MA, Ball RF, Bew MH. First report of Varroa destructor resistance to pyrethroids in the UK. Apidologie. 2002;33(4):357-66.

[R51] Titera D. Vcely zdrave a nemocne [Healthy and diseased bees]. 1^st^ ed. Praha: Brazda; 2017. Czech.

[R52] Traynor KS, Mondet F, de Miranda JR, Techer M, Kowallik V, Oddie MAY, Chantawannakul P, McAfee A. Varroa destructor: A complex parasite, crippling honey bees worldwide. Trends Parasitol. 2020 Jul;36(7):592-606. 32456963 10.1016/j.pt.2020.04.004

[R53] Valizadeh P, Guzman-Novoa E, Goodwin PH. Effect of immune inducers on Nosema ceranae multiplication and their impact on honey bee (Apis mellifera L.) survivorship and behaviors. Insects. 2020 Aug 26;11(9):572. 32858847 10.3390/insects11090572 PMC7563691

[R54] van den Heever JP, Thompson TS, Curtis JM, Ibrahim A, Pernal SF. Fumagillin: An overview of recent scientific advances and their significance for apiculture. J Agric Food Chem. 2014 Apr 2;62(13):2728-37. 24621007 10.1021/jf4055374

[R55] van den Heever JP, Thompson TS, Curtis JM, Pernal SF. Determination of dicyclohexylamine and fumagillin in honey by LC-MS/MS. Food Anal Methods. 2015;8(3):767-77. 10.1016/j.foodchem.2015.01.111 25722149

[R56] vanEngelsdorp D, Underwood RM, Cox-Foster DL. Short-term fumigation of honey bee (Hymenoptera: Apidae) colonies with formic and acetic acids for the control of Varroa destructor (Acari: Varroidae). J Econ Entomol. 2008 Apr;101(2):256-64. 18459386 10.1603/0022-0493(2008)101[256:sfohbh]2.0.co;2

[R57] Vlogiannitis S, Mavridis K, Dermauw W, Snoeck S, Katsavou E, Morou E, Harizanis P, Swevers L, Hemingway J, Feyereisen R, Van Leeuwen T, Vontas J. Reduced proinsecticide activation by cytochrome P450 confers coumaphos resistance in the major bee parasite Varroa destructor. Proc Natl Acad Sci U S A. 2021 Feb 9;118(6):e2020380118. 33547243 10.1073/pnas.2020380118 PMC8017976

[R58] Wilfert L, Long G, Leggett HC, Schmid-Hempel P, Butlin R, Martin SJ, Boots M. Deformed wing virus is a recent global epidemic in honeybees driven by Varroa mites. Science. 2016 Feb 5;351(6273):594-7. 26912700 10.1126/science.aac9976

[R59] Williams GR, Sampson MA, Shutler D, Rogers RE. Does fumagillin control the recently detected invasive parasite Nosema ceranae in western honey bees (Apis mellifera)? J Invertebr Pathol. 2008 Nov;99(3):342-4. 18550078 10.1016/j.jip.2008.04.005

